
JOURNAL INFORMATION
==============================
NLM Title Abbreviation: J Child Orthop
Journal ID: J Child Orthop
NLM Journal ID: 101313582

Journal of Children's Orthopaedics

ISSN: 1863-2521
EISSN: 1863-2548
Publisher: SAGE Publications

ARTICLE INFORMATION
==============================
PMCID: PMC4353818
DOI: 10.1007/s11832-015-0644-1
Article ID: 644
Article version: 1
Subjects: Author Index

Author Index EPOS, 2015, France

Electronic publication date: 2015 Feb 26
Print publication date: 2015 Apr
Volume: 9
Issue: Suppl 1
First page: 1
Last page: 7
Copyright: © The Author(s) 2015
License: This article is distributed under the terms of the Creative Commons Attribution License which permits any use, distribution, and reproduction in any medium, provided the original author(s) and the source are credited.
License URL: https://creativecommons.org/licenses/by/4.0/

issue-copyright-statement: © The Author(s) 2015

==============================

Abbott, Matthew EP5
Abdelaziz, Tarek OP85
Abdelrazek Ahmed, Amin OP83 EP4
Abdul Rahaman, Siti Hauzah EP34
Abelin-Genevois, Kariman OP48
Abizanda, Gloria OP27
Abood, Ahmed EP103
Abousamra, Oussama EP68 OP13
Aboussamra, Oussama OP16
Abramovic, Dusan OP38
Abu Amara, Saad EP26
Accadbled, Franck EP19 OP14 EP74
Adapala, Naga Suresh OP93
Agranovich, Olga OP86
Ahmad, Mubashshar EP127
Ahn, Tae Young EP88
Aird MBBS, MD, FRCS (Orth)
Akbarnia, Behrooz OP68
Akceylan, Anil OP43
Akkena, Sudheer EP127
Akpancar, Serkan EP106
Al Khoury Salem, Hassan EP120
Al Khoury salem, Hassan EP121
Al Khoury salem, Hassan EP118
Alanay, Ahmet OP68
Alderson, Lucy OP3
Ali, Farhan EP127
Alison, Marianne OP74
Allam, Ahmad EP6
Alves, Cristina EP57 OP66 OP45
Anastasopoulos, John EP104
Andras, Lindsay PP15 OP20 OP19 OP53 OP58 EP69
Andras, Lindsay M. EP125
Andreacchio, Antonio EP13
Angelliaume, Audrey EP73
Anticevic, Darko EP114
Antuña, Samuel EP37
Arkader, Alexandre EP116
Armon, Kate EP44
Arnold, Christian EP107
Ashby, Elizabeth OP72 OP70
Assi, Ayman EP41 EP43 EP112 EP42 EP111
Astone, Kristina EP54
Aurore, Bouty EP73
Authier, Guillaume OP49
Authier, Guillaume OP98
Bachy, Manon EP115
Badkoobehi, Haleh OP53 OP58
Bae, Donald S. OP84
Bae, Jung Yun EP10
Baindurashvili, Alexey EP102
Baindurashvili, Alexei OP21
Baindurashvili, Alexey OP86
Bakouny, Ziad EP112 EP111
Balacó, Inês EP57
Balasubramanian, Sriram OP55
Baldwin, Keith EP33
Bar-On, Elhanan EP2
Barbier, Dominique EP15
Barrett, Kody OP20 OP19
Bartley, Carrie OP73
Bastrom, Tracey OP73
Baumgart, Rainer EP75 EP77 OP33
Baunin, Christiane OP14
Baur-Melnyk, Andrea EP72
Bayhan, Ilhan A EP68 OP62 OP16
Beaty, James PP5
Becker, Taly EP2
Berger, Steffen EP122
Bernstein, Robert OP69
Bezdikian, Aren Joe EP111
Bin, Kim OP52 OP49
Birke, Oliver OP44
Bittencourt, Simone EP22
Bixby, Elise OP15 EP54
Björk, Lars EP82
Black, Sheena OP22
Blondel, Benjamin OP47 EP11
Blumetti, Francesco EP22 OP12
Blumstein, Gideon EP123
Bober, Micheal B OP16
Bohl, Michael OP18 EP129
Boissière, Louis EP73
Bollini, Gérard OP98
Boorman-Padgett, James PP13
Bouchard, Maryse OP57
Boulay, Christophe OP98
Bowen, J. Richard EP36
Bowman, Christine OP20 OP19
Brabham, Case OP93
Bradish, Christopher OP6
Bradshaw, David OP44
Brdar, Radivoj OP38
Breyer, MD, Sandra OP91
Bricoteau, Maxime EP29
Brink, Rob EP79
Briot, Jerome OP48

Broom, Alexander EP116 OP20 OP19 OP53
Broom, Alexander M. EP125
Brunier, Marion OP61
Bruno, Dohin EP118
Buck, Florian OP59
Bucklaev, Dmitriy EP102
Bukva, Bojan OP38
Burgemeister, Karen EP107
Burgess, Jamie OP90

Caff, Giuseppe EP25
Cage, Matthew OP22
Campbell, Robert M. OP55 EP33
Camus, Dimitri EP81
Canavese, Federico PP4
Canavese, Federico OP60
Canavese, Federico EP13 OP45
Castanier, Elisabeth OP98
Castelein, René EP79
Castro, João Diogo EP57
Ceroni, Dimitri EP119 OP100
Chabrol, Brigitte OP98
Chalopin, Antoine OP47
Charles, Philippe OP60
Chatzipantelis, Stefanos EP89
Cheesman BSc MBChB MRCS(Eng), Caroline L EP21
Chen, Po Jung EP78
Chen, Elena OP93
Cheng, Tegan PP3 OP32 OP26
Cheng, Jack EP79
Chessman BSc MBChB MRCS(Eng), Caroline OP40
Cheung, Kenneth OP68
Cheverda, Andriy EP39
Chiaia, Theresa EP51
Cho, Tae-Joon EP94 EP99 EP97 EP91 EP96 OP10 OP11 EP76 OP46 EP98 EP93
Choi, In Ho EP94 EP99 EP97 EP91 EP96
Choi, Paul PP15
Choi, In Ho OP10 OP11 EP76 OP46
Choi, Paul OP58
Choi, In Ho EP98 EP93
Chu, Alice EP67
Chu, Stephanie EP69
Chua, Matthew OP23
Chung, Chin Youb EP94 EP99 EP95 EP97
Chung, Myung Ki EP97
Chung, Chin Youb EP91
Chung, Myung Ki EP96
Chung, Chin Youb EP96
Chung, Myung Ki OP11
Chung, Chin Youb OP11 OP46
Chung, Myung Ki EP98
Chung, Chin Youb EP98
Chung, Myung Ki EP93
Chung, Chin Youb EP93
Church, Chris OP13
Cobanoglu, Mutlu PP6
Coleman-Wood, Krista EP63
Colo, Dino EP79
Cooperman, Daniel OP18 OP7 OP30
Cordasco, Frank PP13 EP51 EP27
Corominas, Laura EP45
Cortez, Leonardo EP22
Cosgrove, Aidan EP85
Cosma, Dan OP66
Covaro, Augusto EP64
Cravino, Mattia EP13
Crawford, Haemish OP69
Crompton, Thomas EP3
Cullu, Emre PP6
Cundy, Peter EP24
Cunin, Vincent EP26
Cyr, Micaela EP125

Dabney, Kirk OP13
Daluiski, Aaron EP53
Dana, Caroline EP115
Danino, Barry OP67
Dapic, Tomislav EP114
Dare, David PP9 EP27
Dawicki, Erin OP20 EP69
De Rosa, Vincenzo EP13
Degueldere, Vincent EP28
Deja, Mario EP107
Dela Torre, Katrina EP60
Delcourt, TIphanie EP83
DelPizzo, Kathryn EP31
DeMille, Polly EP51
Demirkiran, Gokhan OP68
Dempsey, Molly OP90
Deo, Nikita OP32
Derevianko, Denis EP102
Dhouib, Amira OP100
Di Gennaro, Giovanni Luigi PP2
Diaz-Gallardo, Paula OP8 EP124
Dimeglio, Alain OP60
Dimitriadis, Dimitris EP89
DiNardo, Anthony PP18
Ditro, Colleen OP62
Djamel, Louahem Msabah OP79
Djuricic, Goran OP38
Docquier, Pierre-Louis EP29
Docquier, Pierre-Louis EP1
Dodwell, Emily EP53 OP15 OP45 EP52 EP54
Doederlein, Leonhard OP9
Dohin, Bruno EP120
Dohin, Bruno EP121
Domos, Gyula EP117
Dotor, Javier EP65
Dow, Matthew OP15
Drissi, Hicham OP93
Drury, Rosalyn EP126
Duart, Julio EP108
Duart-Clemente, Julio EP65 OP27
Dubois, Rémi OP61
Ducic, Sinisa OP38
Dussa, Chakravarthy U OP9

Eamsobhana, Perajit OP76
Eastwood, Deborah M OP6
Eastwood, Deborah OP3 OP66 EP59
Eibl, Dominik EP72
Eid, Tony EP41
El-Hawary, Ron OP51
Elbaum, Robert EP28 EP29
Er, Mehmet S OP62
Ernat, Justin OP88 OP99
Estivalezes, Erik OP48
Ey, Anna OP66
Ey Batlle, Ana Maria OP2 EP64

Fabricant, Peter PP13 EP27
Farcetta Júnior, Fernando EP22
Farhang, Kathleen EP9
Farr, Sebastian OP87 OP84
Farrow, Lutul PP7
Farsetti, Pasquale EP61
Fassier, Francois OP72 OP70
Faulks, MSR, RN, CNS, Shawne OP5
Faulks, MSR, RN, CNS,, Shawne OP1

Felton, CPO, Kevin OP1
Ferguson, John OP68
Ferrero, Emmanuelle OP50 OP74 EP17
Fields, Kara EP31 EP32
Finidori, Georoges EP83
Fiocco, Marta EP105
Firth, Greg EP80
Fishkin, Michael EP12
Fitzpatrick, Kelly OP88
Fletcher, Nicholas OP20 OP19
Fliesenberg, Katharina EP107
Flynn, John M. EP125 EP33
Formentin, Cristina OP63
Foss, Olav Andreas PP8
Fowers, Cody PP16 OP30
Franklin, Don PP5
Frontera- Juan, Guillem EP45
Frontera-Juan, Guillem EP108
Fujino, Marcelo EP22 OP12

Gal, M.D., prof., Petr EP90
Galvez, Elena EP37
Ganger, Rudolf OP87
Gantelius, Stefan EP82
García-Mata, Serafín EP108
Garg, Sumeet EP125
Garner, Matthew OP15
Gausden, Elizabeth PP10 EP50
Gebhart, Jeremy OP18 EP129
Geertsema, Dorien EP105
Geith, Tobias EP72
Gelfer, Yael OP66
Gera, Sumanth EP34
Ghanem, Ismat EP41 EP43 EP112 EP42 EP111
Gheen, William OP99
Ghosn, Elias EP41
Gigante, Cosimo OP63
Gilmore, Allison PP7
Giorgi, Hadrien OP98
Girach, Julekha EP44
Girsch, Werner OP87
Glorion, Christophe EP83
Glorion, Christophe EP115
Goldstein, Rachel EP66 EP67 EP123
Goldstein, Rachel Y. EP116
Goldstein, Rachel OP20 OP19
Goldstein, Rachel Y. OP65
Goldstein, Rachel EP69
Gonzalez-Gil, Ana-Belen OP27
Gopinathan, Nirmal Raj EP55
Gosheger, Georg OP37
Gottliebsen, Martin EP103
Granero-Molto, Froilan OP27
Graziano, Jessica EP51
Grebenyuk, Eugeny OP39
Green, Daniel PP13 PP10 PP9 EP49 EP50 EP51 EP52 EP27
Greene, Janelle OP7
Gremark, Ola EP82
Grill, Franz OP67
Grosz, Gabor EP117
Gudmundsson, Paul OP93 OP90
Guk, Iurii EP39
Guney, Ahmet EP113

Ha, Myong Su EP38
Hachache, Bilal EP41
Haddad, Elie EP118
Hamdy, Reggie C. OP72 OP70
Harcke, H. Theodore EP36
Haskel, Jonathan PP10 PP9 EP50
Hattori, Kazuki EP38
Healy, Bitte OP65
Hedequist, Daniel OP69
Heflin, John OP51
Heimkes, Bernhard EP72
Helenius, Ilkka OP56 OP69 OP68
Hemo, Yoram OP66
Henley, John OP13
Hensley, Dana EP7
Hernandez-Soria, Alexia EP53
Herzenberg, John E. OP67
Ho, Christine OP99
Hogue, Grant PP1
Holen, Ketil Jarl PP8
Holmes, Jr., Laurence EP36
Holroyd, Benjamin OP41
Horter, Melanie OP37
Horvath, Nikoletta EP117
Hosny, Gamal EP71
Houdek, Matthew OP94
Huang, Shier-Chieg EP8
Hutchinson, Rachael EP30 EP44
Hwang, Jin Ho EP92

Iacobellis, Claudio OP63
Idzior, Maciej EP78
Iershov, Dmytro OP71
Igah, Ibidumo EP40
Ilharreborde, Brice EP63 OP50 OP74 EP109
Ilharreborde, Brice EP26
Ilharreborde, Brice EP17
Imhauser, Carl PP13
Inan, muharrem PP14
Incesu, mustafa PP14
Ippolito, Ernesto EP61
Ito, Kinya EP38
Ivanov, Stanislav OP21
Izadi, Ebraheim EP40
Izal, Iñigo EP65

Jacquemier, Michel OP98
Jahandar, Hamidreza PP13
Jalanko, Tuomas OP56
James J EP21 OP40
Jerome, Cottalorda OP79
Jo, Byung Chae EP94 EP99 EP91
Jo, ChanHee OP90
Jochymek, M.D., Ph.D., Jiri EP90
Johal, Simran EP126
Joiner, Elizabeth PP15
Jones, Kristofer EP27
Joo, Sun Young EP14
Joukhaider, Nadim OP51
Journeau, Pierre EP15
Jouve, Jean-Luc OP47
Jouve, Jean Luc OP52
Jouve, Jean-Luc OP49
Jouve, Jean Luc OP61
Jouve, Jean-Luc OP98
Jouve, Jean-Luc EP11
Jozwiak, Marek EP78

Kaddaj, Reda OP61
Kafadar, Ibrahim Halil EP113
Kakizaki, Jun OP64 OP82
Kalish, Leslie A. OP84
Kamegaya, Makoto OP64 OP82
Kamiya, Nobuhiro OP93
Kampouroglou, Georgios OP100
Kang, Seungcheol OP4
Karaman, Ibrahim EP113
Karol, MD, Lori OP1
Katsoulas, Dimitrios EP104
Katzel, Mia OP23
Kaufman, Kenton R. EP63
Kawamura, Cátia EP22
Kawamura, Catia OP12
Kay, Robert EP123
Kedem, Paz PP12
Kelly, Bryan EP32
Kelly, Derek PP5

Kemp, Jenny OP3
Kenis, Vladimir OP21
Keskinen, Heli OP56
Khalil, Nour EP112 EP111
Khamaisy, Saker PP12
Khan, Shah Alam EP70
Khandwala, Yash OP73
Kharrat, Khalil EP41
Khmyzov, Sergey OP71
Kilimci, Figen Sevil PP6
Kim, Hui Taek EP88
Kim, Han-Ju EP35
Kim, Jung Ryul OP25
Kim, Harry OP93
Kim, Yeon Soo OP10
Kim, Han-Ju OP34
Kim, Joon Ho OP46
Kim, Harry OP90
Kinchaya-Polishchuk, Tamara EP39
Kiss, Laszlo EP117
Kiss, Sandor EP117
Kjeldgaard Pedersen, Line EP84
Klein, Celine EP83
Kneissel, Michaela OP28 OP26
Knorr, Jorge OP8
Knorr, Gorka EP74
Knorr, Jorge EP124
Koca, Kenan EP106
Kohler, Rémi OP61
Kokkinakis, Michail EP3
Kononovich, Natalia EP15
Korhonen, Katariina OP56
Kothari, Alpesh EP16
Krallis, Panagiotis EP104
Kramer, Ina OP28 OP26
Kreichaty, Gaby EP43
Krengel, Walter OP57
Kubat, Ozren OP51 EP114
Kumar, Ashok EP70
Kuo, Ken N. EP8
Kuruba, Venkatramana EP55
Kwan, Kenny OP68
Kwon, Dae Gyu EP94 EP99 EP97 EP91
Kwon, Soon-Sun EP96
Kwon, Dae Gyu EP96 OP11 EP98
Kwon, Soon-Sun EP93
Kwon, Dae Gyu EP93
Köse, Nusret OP43
Larson, A Noelle EP87 OP54
Larson, Noelle EP17
Larson, A Noelle EP86
Lascombes, Pierre EP15
Launay, Franck EP81
Lebel, David OP45
Lee, Kyoung Min EP94
Lee, Sang Hyeong EP94
Lee, Seung Yeol EP94
Lee, Kyoung Min EP99
Lee, Sang Hyeong EP99
Lee, Seung Yeol EP99
Lee, In Hyeok EP95
Lee, Kyoung Min EP95
Lee, Jehee EP95
Lee, Sang Hyeong EP97
Lee, Kyoung Min EP97
Lee, Seung Yeol EP97
Lee, Ji-Ho EP35
Lee, Kyoung Min EP91
Lee, Sang Hyeong EP91
Lee, Seung Yeol EP91
Lee, Kyoung Min EP96
Lee, Sang Hyeong EP96
Lee, Shiela EP30
Lee, Dong Yeon OP10
Lee, Ji-Ho OP34
Lee, Kyoung Min OP11
Lee, Sang Hyeong OP11
Lee, Seung Yeol OP11
Lee, Kyoung Min EP98
Lee, Sang Hyeong EP98
Lee, Kyoung Min EP93
Lee, Sang Hyeong EP93
Lefèvre, Yan EP73
Lehman, Wallace EP67
Lennon, Nancy OP13
Leon, Viktoria OP44
Levack, Ashley EP31
Li, Ryan OP36
LI, Xu OP97
Li, Zhao OP29
Lim, Kevin EP34
Limpaphayom, Noppachart EP20
Ling, Tah Pu EP57
Lins Neves, Daniella EP22 OP12
Little, David PP3 OP28 OP32 OP44 OP26 EP46
Liu, Raymond EP9 PP7 PP16 OP31 OP18 OP7 OP36 OP30 EP129
Lloyd, Claire EP3
Lofterød, Bjørn EP23
Lopes, José Augusto EP22 OP12
Loucheur, Naïma OP61
Lukkarinen, Heikki OP56
Luo, T David EP86
Luther, Herman OP19 EP69
Lutz, Nicolas OP81
Lyman, Stephen EP53 EP52

Ma, Ruixue EP128
Mackenzie, William PP18
Mackenzie, William G OP62 OP16
Maizen, Claudia EP40
Malagelada, Francesc EP80
Mallet, Cindy EP109
Mansour, Mounira PP4 EP13
Marengo, Lorenza EP13
Marky, Chris PP18
Martsynyak, Stepan EP39
Mascard, Eric EP115
Massaad, Abir EP112
Matos, Gabriel EP57
Mayer, Stephanie EP32
Mayer, Oscar H. EP33
Mayet, Saadia EP80
Mazda, Keyvan OP50 OP74 EP109
McCarthy, Moira PP13
McCarthy, Richard OP53
McCarthy, Moira EP51 EP52 EP27
McCullough, Lynn OP53
McDonald, Michelle OP28
McDonough, Joseph EP33
McIntosh, Amy OP54
McKie, Janay EP7
McLawhorn, Alexander EP31
Medina, Juan Manuel EP103
Meinardi, Joris E EP105
Menekseoglu, Nuri EP106
Mercado, Eyal EP2
Metcalfe, James OP41
Meyer, Russell PP10
Meyrignac, Olivier OP14
Mikulec, Kathy OP26
Miller, Andy PP11
Miller, Freeman OP13 OP62 OP16
Millis, Michael OP20 EP69
Mintz, Douglas OP15
Mo, Yueqiang EP128
Moisan, Alice EP125
Moller-Madsen, Bjarne EP103
Mong, Andrew EP33
Monsell BCh, MSc, PhD, FRCS, F, Fergal P OP40

Monsell MB BCh, MSc, PhD, FRC, Fergal P EP21
Montpetit, Kathleen OP72 OP70
Mora, Gonzalo EP65
Morais Filho, Mauro Cesar EP22 OP12
Morakis, Emmanuel EP127
Morakis, Emanouil EP104
Moraleda, Luis OP77 EP37
Moran, Steven OP94
Morin, Christian OP47
Morita, Mitsuaki OP64 OP82
Morris, William PP16 OP30
Morse, Alyson OP28
Mosow, Nadine OP91
Mueske, Nicole OP23 OP65
Munns, Craig PP3 OP26
Munzer, Caroline EP74
Musielak, Bartosz EP78
Møller-Madsen, Bjarne EP84
Nadler, Robert EP126
Nakamura, Naoyuki OP96
Nance, Jennifer EP7
Nance, Michael EP33
Naoum, Elias EP42
Narayanan, Unni OP67
Nawabi, Danyal EP51
Neal, David OP93
Newton, Peter OP73
Nguyen, Cynthia PP7
Nguyen, Joseph PP10
Nicholson, Allen OP7
Nicolas, Nicolas EP43
Niiler, Tim OP13
Nikolajsen, Lone EP84
Nishnianidze, Tristan EP68 OP62 OP16
Niziol, Rafal EP110
Nnadi, Colin OP68
Noel, Brigitte EP28 EP29
Norman-Taylor, Fabian EP3
Nunes da Paz, Stefanie EP122

O'Brien, Sean PP11
O'Brien, Jack OP93
Obeid, Ibrahim OP52
Ocal, Mehmet Kamil PP6
Odent, Thierry OP69
Oikawa, Yasuhiro OP64
Ömeroglu, Hakan OP43
Ondrus, M.D., Ph.D., Simon EP90
Oner, Mithat EP113
Osateerakun, Phatcharapa EP20
Osman, Mohamed OP89
Otsuka, Takanobu EP38
Ounpuu, Sylvia OP65
Øye, Christian PP8

Pace, J. Lee EP116
Pace, James Lee OP23
Pagliazzi, Gherardo PP2
Pagnotta, Gaetano OP66
Palmen, Nina-Kristin OP92
Pan, Ting-Jung EP53 EP52
Panitch, Howard EP33
Pannier, Stephanie EP83
Park, Moon Seok EP94 EP99 EP95 EP97
Park, Paul EP9
Park, Kwang-Won EP35
Park, Moon Seok EP91 EP96
Park, Kwang-Won OP34
Park, Moon Seok OP11
Park, Soo-Sung OP4
Park, Moon Seok OP46 EP98 EP93
Parratt, Tim M OP6
Pashenko, Andrey OP71
Patel, Nimesh OP95 EP58
Pavone, Vito EP25
Peacock, Lauren OP26
Pei, Xinhong EP128
Peltier, Emilie OP47 OP52 EP11
Penneçot, Georges EP109
Pereira, Bruno PP4
Pereira, Bruno OP60
Perlman, Stephanie PP11
Pesenti, Sébastien OP47
Pesenti, Sébastien OP52
Pesenti, Sebastien OP49
Pesenti, Sébastien EP11
Peters, James OP55
Petratos, Dimitrios EP104
PG Zaini, DK sa'diah EP34
Philippe, Mazeau OP79
Phillips, Jonathan OP69
Phoon, Christopher EP46
Pineault, Kevin OP13
Pirsa, Matea EP114
Planka, M.D.,prof., Ladislav EP90
Podeszwa, David OP99
Polirsztok, Eva OP49
Politorszk, Eva OP48
Pomero, Vincent OP49
Pomero, Vincent OP98
Pomero, Vincent EP11
Pontailler, Jean-Roger EP73
Pontén, Eva EP82
Popkov, Dmitry OP39
Popkov, Arnold OP39
Popkov, Dmitry OP35
Popkov, Arnold OP35
Popkov, Dmitry EP15
Poudel, Rishi Ram EP70
Poul, Jan EP56
Presedo, Ana EP109
Price, Charles EP60
Prof. Dr. Krauspe, Ruediger OP92
Prof. Dr. Westhoff, Bettina OP92
Prosper, Felipe OP27
Protopapa, Evangelia EP110
Pruszczynski, Blazej EP36 OP42

Radler, Christof OP66
Radlovic, Vladimir OP38
Rahbek, Ole EP84 EP103
Ramachandran, Manoj EP80 EP103
Ramseier, Leonhard OP59
Rathjen, MD, Karl OP1
Razak, Arif EP127
Rebolledo, Brian EP27
Rehm, Andreas EP126 EP101
Reighard, Fredrick OP73
Rethlefsen, Susan EP123
Ricciardi, Benjamin EP32 EP60
Riccio, Anthony OP88 OP22 OP99 EP5 EP7
Riccioli, Maria EP25
Richards, MD, B. Stephens OP1 OP5
Ridderbusch, MD, Karsten OP91
Roaten, John EP125
Robinson, Elaine EP85
Rocak, Karel EP56
Rodeo, Scott PP9
Rodriguez de la Rubia, Enrique OP78
Rodriguez-Goñi, Maria-Victoria EP65
Roedl, Robert OP37
Rogalski, Matthias EP107
Rogers, Kenneth J EP68
Rogers, Kenneth PP18
Rogers, Kenneth J OP62 OP16
Romanenko, Konstantin OP71
Roposch, Andreas EP110 OP66

Rosenthal, Dietmar OP92
Rosskopf, Andrea OP59
Rousset, Marie PP4
Rousset, Marie OP60
Rousset, Marie EP13
Runner, Robert OP20 OP19
Rupprecht, MD, Martin OP91
Ryan, Deirdre OP65
Rödl, Robert OP67

Sabbah, David OP50
Sabet, Nagy EP47
Sager, Brian PP1
Saghbini, Elie EP42
Saisu, Takashi OP64 OP82
Sakuma, Eisuke EP38
Sala, Debra EP67
Salabi, Vincent EP62
Sales de Gauzy, Jérôme EP19 OP14
Sales de Gauzy, Jerome OP8
Sales de Gauzy, Jérôme OP47
Sales de Gauzy, Jerome OP48
Sales de Gauzy, Jérôme EP74
Sales de Gauzy, Jerome EP124
Sales de Gauzy, Jérôme EP73
Salom, Marta EP108
Salon, Arielle EP83
Salon, Arielle EP115
Salvatore, Christine PP11
Salvo, Davide OP100
Samba, Antoine PP4
Samba, Antoine OP60
Samba, Antoine EP13
Sampath Kumar, Venkatesan EP70
Sanders, James OP7
Sanghrajka, Anish P. EP30
Sanghrajka, Anish EP44
Sanpera, Ignacio EP45 OP78
Sanpera, Ignacio Jr EP108
Santos, Carlos Alberto EP22
Sanz-Ramos, Patricia EP65
Sarikaya, Ilker PP14
Sarker, Shahrier EP101
Sauret, Christophe EP112 EP111
Saus-Sarrias, Carles EP45
Sawyer, Jeffrey PP5 EP125
Saxena, Akshay Kumar EP55
Scandella, Marino EP74
Schade BSc, Alexander T OP40
Scher, David PP12
Schiedel, Frank OP37
Schindeler, Aaron PP3 OP28 OP32 OP26
Schlösser, Tom EP79
Schueler, Beth EP86
Schuller, Sebastien OP60
Sciberras, Nadia OP41
Sebaaly, Amer EP41
Sebag, Guy OP74
Sees, Julieanne EP48
Segawa, Yuko OP64 OP82
Segev, Eitan OP67
Seker, Ali PP14
Seo, Sang Gyo OP10
Sessa, Giuseppe EP25
Sestokas, Anthony PP18
Sever, Ronen EP12
Shabtai, Lior EP116 OP67
Shah, Suken A EP68
Shaha, James OP22
Shaha, Steven OP22
Sheha, Evan EP31
Shi, Qiang OP97
Shin, Yong-Woon PP10
Shin, Chang Ho OP46
Shin, Yong-Woon EP49
Shirai, Yasuhiro EP38
Shutov, Roman EP15
Sim, Franklin OP94
Sim, Francis EP101
Simon, Anne-Laure EP63 EP109 EP17
Sink, Ernest EP31 EP32 EP60
Skaaret, Ingrid EP23
Skaggs, David PP15 OP19 OP51 OP53
Skaggs, Kira OP58
Skaggs, David OP58
Skalli, Wafa EP112 EP111
Slaba, Sami EP43
Smigovec, Igor EP114
Smith, Richard PP5
Soldado, Francisco OP8 EP124
Sommerfeldt, Dirk OP80
Song, Hae-Ryong EP35 OP34
Song, Mi Hyun EP76
Sousa, Ted EP116 PP15
Spence, David EP85 PP5
Sponseller, Paul OP69
St George, Justine OP44 EP46
Stans, Anthony EP87 OP54 OP94 OP69 EP86
Starr, Adam PP1
Stebbins, Julie EP16
Steffen, Jean Sébastien OP52
Steib, Jean-Paul OP60
Steiger, Christina EP119
Stephens, Byron PP5
Stewart, David EP39
Stilli, Stefano PP2
Stitzman, Melisa OP2
Stitzman Wengrowicz, Melisa EP64
Stohr, Kuldeep EP110
Stone, Kyle PP13
Streit, Jonathan EP129
Struijs, Peter AA OP75
Study Group, Growing Spine OP53
Stücker, Prof., Ralf OP91
Su, Alvin OP54
Sudesh, Pebam EP55
Suh, Seung Woo EP92
Suleiman, Firas EP18
Sung, Ki Hyuk EP94 EP99 EP97 EP91 EP96 OP11 EP98 EP93
Sutton, Fran EP30
Swider, Pascal OP48
Synder, Marek OP42
Szoke, George EP117
Sá Cardoso, Pedro EP57

Tanaka, Reiko OP64 OP82
Taylor, Clare OP41
Tennant, Sally EP100
Terebessy, Tamas EP117
Terjesen, Terje EP23
Testa, Gianluca EP25
Thacker, Mihir PP18
Thein, Ran PP12
Theologis, Tim EP16
Thomas, Joanna EP44
Thomson, Jeffrey OP65
Thoveson, Alec OP93 OP90
Thévenin Lemoine, Camille EP19
Tiwari, Vivek EP70
Tolo, Vernon PP15
Tomaszewski, Ryszard PP17 OP24
Tong, Yubing EP33
Trehan, Samir EP49
Tretow, Henning OP37
Trisolino, Giovanni PP2
Trofimova, Svetlana EP102
Truong, Walter OP45
Tsitskaris, Konstantinos OP6
Tsukagoshi, Yuta OP64

Udupa, Jayaram EP33
Uppstrom, Tyler PP10 PP9 EP49 EP50
Urbasek, Karel EP56
Ursei, Monica EP74

Vaikute, Rima OP41
Valaikaite, Raimonda OP100
Valencia, Maria OP77
Valentí-Nin, Juan-Ramon OP27
Van De Sande, Michiel A.J. EP105
van Stralen, Marijn EP79
Vandergugten, Simon EP1
Vena, Giuseppe EP61
Ventura Gómez, Norberto OP2
Ventura Gómez, Norbert EP64
Vial, Julie OP14
Vicente-Pascual, Mikel EP65
Viehweger, Elke OP49
Viehweger, Elke OP98
Viehweger, Elke EP11
Vilalta Vidal, Imma OP2
Vilalta Vidal, Inmaculada EP64
Vincken, Koen EP79
Virostek, CPO, Donald OP1
Vital, Jean Marc OP52
Vlachou, Maria EP89
Vogt, Bjoern OP37

Wada, Ikuo EP38
Wagner, Eric OP94
Wakabayashi, Kenjiro EP38
Wang, Cheng-Wei EP8
Wang, Ting-Ming EP8
Wang, Wei-Hsun EP33
Wansbrough, Guy OP95 EP58
Warner, Stephen PP11
Waters, Peter M. OP84
Weigl, Daniel EP2
Weinberg, Doug EP9
Weinberg, Douglas OP31 OP18
Widmann, Roger PP11 PP12 OP15 EP49 EP54
Wiegerinck, Johannes OP75
Wientroub, Shlomo OP66 OP67 EP12
Williams, Daniel OP6
Wilson, Neil OP89
Wilson, Lance OP95 EP58
Wilson, Philip EP5
Wimberly, Robert OP88 OP22 OP99 EP5 EP7
Witbreuk, Melinda OP17
Wolff, Stéphane OP47
Wren, Tishya EP123 OP23 OP65
Wu, Kuan-Wen EP8
Wu, Zhenkai OP29
Wu, Ivy EP33

Xie, Katherine OP30

Yamaguchi, Ryosuke OP93
Yang, Jae Hyuk EP92
Yaniv, Moshe EP12
Yared, Fares EP111
Yasin, Mohamad OP51
Yasmeh, Pauline OP65
Yaszay, Burt OP73
Yaygingul, Rahime PP6
Yazici, Muharrem OP68
Yellin, Joseph EP125
Yildirim, Enis PP14
Yildiz, Cemil EP106
Yoo, Won Joon EP94 EP99 EP97 EP91 EP96 OP10 OP11 EP76 OP46 EP98 EP93
Yorgova, Petya EP68
Youn, Kibeom EP95
Young, Ernest EP87
Yuh, Roger PP16 OP30

Zacher, Joseph EP107
Zaslow, Tracy OP23
Zavatsky, Amy EP16
Zgoda, Marcin OP89
Ziebarth, Kai EP122
Ziegler, Christian EP72
Zilkens, Christoph OP92
Zyma, Andrii EP39

Open Access

This article is distributed under the terms of the Creative Commons Attribution License which permits any use, distribution, and reproduction in any medium, provided the original author(s) and the source are credited.

